# Investigation of direct inkjet-printed versus spin-coated ZrO_2_ for sputter IGZO thin film transistor

**DOI:** 10.1186/s11671-019-2905-2

**Published:** 2019-03-05

**Authors:** Wei Cai, Honglong Ning, Zhennan Zhu, Jinglin Wei, Shangxiong Zhou, Rihui Yao, Zhiqiang Fang, Xiuqi Huang, Xubing Lu, Junbiao Peng

**Affiliations:** 1 Institute of Polymer Optoelectronic Materials and Devices, State Key Laboratory of Luminescent Materials and Devices, South China University of Technology, Guangzhou, 510640 China; 2 State Key Laboratory of Pulp and Paper Engineering, South China University of Technology, Guangzhou, 510640 China; 3 Gu’an New Industry Demonstration Zone, Langfang, 065500 Hebei People’s Republic of China; 4 Institute for Advanced Materials and Guangdong Provincial Key Laboratory of Quantum Engineering and Quantum Materials, South China Normal University, Guangzhou, 510006 China

**Keywords:** Oxide dielectric, Direct inkjet printing, Interface, Bias stress stability

## Abstract

In this work, a low leakage current ZrO_2_ was fabricated for sputter indium gallium zinc oxide (IGZO) thin-film transistor using direct inkjet-printing technology. Spin-coated and direct inkjet-printed ZrO_2_ were prepared to investigate the film formation process and electrical performance for different process. Homogeneous ZrO_2_ films were observed through the high-resolution TEM images. The chemical structure of ZrO_2_ films were investigated by XPS measurements. The inkjet-printed ZrO_2_ layer upon IGZO showed a superior performance on mobility and off state current, but a large *V*_th_ shift under positive bias stress. As a result, the TFT device based on inkjet-printed ZrO_2_ exhibited a saturation mobility of 12.4 cm^2^/Vs, an I_on_/I_off_ ratio of 10^6^, a turn on voltage of 0 V and a 1.4-V *V*_th_ shift after 1-h PBS strain. Higher density films with less oxygen vacancy were responsible for low off state current for the printed ZrO_2_ device. The mechanism of deteriorated performance on PBS test can be ascribed to the In-rich region formed at the back channel which easily absorbs H_2_O and oxygen. The absorbed H_2_O and oxygen capture electrons under positive bias stress, serving as acceptors in TFT device. This work demonstrates the film formation process of direct inkjet-printed and spin-coated oxide films and reveals the potential of direct inkjet-printed oxide dielectric in high-performance oxide TFT device.

**Electronic supplementary material:**

The online version of this article (10.1186/s11671-019-2905-2) contains supplementary material, which is available to authorized users.

## Background

Metal oxide dielectrics have recently emerged as promising alternatives to SiO_2_ and SiN_*x*_ in thin-film transistors (TFTs) owing to their superior properties, including high capacitance, low defect states, and large band gap which leads to high mobility and low off current [1–3]. For these reasons, oxide dielectrics fabricated by vacuum process are widely studied in displays, sensor arrays, and driving circuits [4]. Meanwhile, the solution process has also received remarkable attention because of the advantage of low cost for large-scale fabrication including spin coating, inkjet printing, spray coating, and slit coating [5, 6]. Among these, direct inkjet printing is the most promising method which can achieve patterned films without photolithography. However, TFT devices fabricated by the inkjet-printing process exhibit inferior electrical performances compared to the vacuum-processed ones. Direct inkjet-printing metal-oxide films face serious problems: (1) the uncontrollable spreading of oxide precursor on the substrate due to the difference of surface energy of the fluid and substrate and (2) the compatibility of printed oxide dielectrics with semiconductor [7].

The film formation process of solution-processed dielectric film has significant influence on electrical property. The spin-coating method as an established technique is widely used in solution-processed TFT. The leakage current density of spin-coated oxide dielectric is usually lower than 10^−6^ A/cm^2^ at 1 MV/cm, and the breakdown electric field is more than 2 MV/cm. Saturation mobility of TFT based on coated oxide dielectric is around 10 cm^2^/Vs. However, for printed oxide dielectric, the leakage current density is about two orders of magnitude higher than that for coated oxide film (>10^− 4^ A/cm^2^ at 1 MV/cm) and saturation mobility is lower than 5 cm^2^/Vs. Few reports have made comparison of inkjet-printed dielectric films with spin-coated films especially on the film formation process. Density, surface roughness and homogeneity of dielectric films are the most important factors related to the electrical performance of TFT [8]. Moreover, the interface between gate insulator and semiconductor also plays a key role for the solution process TFT [9]. A comprehensive study on inkjet-printed oxide dielectrics is of great value to better understand this promising technique.

In this paper, we prepared high-quality ZrO_2_ films with favorable surface appearance and excellent electrical performance by both coating and printing method and investigated the electrical effect applied in sputtered indium gallium zinc oxide (IGZO) TFT [10, 11]. The film formation process of the spin-coating and direct printing methods is compared. The spin-coating method is dominated by centrifugal force leading to uniform but dispersive distribution of molecules while the inkjet-printing process depends on fluid dynamics. According to XPS and IV test, inkjet-printed ZrO_2_ film (double layers) had less oxygen vacancies compared with the spin-coated one. Increasing printed layers of ZrO_2_ films can fill the holes and vacancies created by unsteady flow of precursor spreading on the substrate, contributing to less defect and superior uniformity. the direct inkjet-printed ZrO_2_ film for sputtered IGZO has lower leakage current density, higher mobility, larger on/off ratio, and larger *V*_th_ shift under positive bias stress than the spin-coated ZrO_2_-TFT. The In-rich region formed at the back channel of inkjet-printed ZrO_2_ TFT is responsible for worse stability since water molecules and oxygen in the air can easily be absorbed in under positive bias stress, consuming electrons from the IGZO layer. It reveals that the direct inkjet-printing technique is able to fabricate high-density oxide dielectric but the interface defect should be well controlled to avoid electrical instability.

## Methods

### Materials

The ZrO_2_ solution was synthesized by dissolving 0.6 M ZrOCl_2_·8H_2_O in a 10 ml mixture solvent of 2-methoxyethanol (2MOE) and ethylene glycol with a ratio of 2:3 to attain a certain surface tension of precursor. The solution was stirred at 500 r/min at room temperature for 2 h, followed by aging for at least 1 day. For ozone UV treatment process, a 100-W UV lamp with 250 nm wavelength was used to irradiate the indium tin oxide (ITO) substrate cleaned by isopropyl alcohol and deionized water. Subsequently, ZrO_2_ films were formed by spin coating or direct inkjet-printing process. The coating process was carried out with a speed of 5000 rpm for 45 s, while the drop space and nozzle temperature are 30 μm and 30 °C for the printing process. ZrO_2_ films were annealed at 350 °C under atmospheric environment for 1 h. 10-nm-thick IGZO was then grown by direct current pulsed sputtering method with a pressure of 1 mTorr (oxygen:argon = 5%) and patterned by shadow mask. IGZO was annealed at 300 °C for 1 h to reduce the defect in the film. The channel width and length were 550 μm and 450 μm; thus, the width/length ratio was 1.22. Finally, Al source/drain electrodes with 150-nm thickness were deposited by direct current sputtering at room temperature.

### Instruments

X-ray photoelectron spectroscopy (XPS) measurements were carried out to investigate the chemical structure in oxide semiconductors performed by ESCALAB250Xi (Thermo-Fisher Scientific, Waltham, MA, USA) at a basic pressure of 7.5 × 10^−5^ mTorr. The cross-sectional transmission electron microscopy (TEM) images were measured by JEM-2100F (JEOL, Akishima, Tokyo, Japan) and the results of electronic differential system (EDS) mapping scan were analyzed by Bruker (Adlershof, Berlin, Germany) to investigate the element distribution. Under the dark condition and air at RT, capacitance–voltage curves were measured by an Agilent 4284A precision LCR Meter (HP, USA). To measure the transfer characteristics of IGZO TFT and leakage current density curves, we used Agilent 4156C precision semiconductor parameter analyzer. Transfer characteristics were measured by a gate voltage sweeping from − 5 to 5 V with a drain voltage of 5 V. We calculated the field effect mobility using the measured transfer curve and the following equation:

1 $$ {I}_{\mathrm{DS}}=\frac{W\mu {C}_{\mathrm{i}}}{2L}{\left({V}_{\mathrm{GS}}-{V}_{\mathrm{th}}\right)}^2 $$

where I_DS_, C_i_, μ, W, L, V_GS_, and V_th_ are the drain current, capacitance of the gate dielectric per unit area, saturation mobility, channel width, channel length, gate voltage, and threshold voltage, respectively. The dielectric constant is calculated by equation as follow:

2 $$ {\varepsilon}_{\mathrm{r}}=\frac{C\cdotp d}{\varepsilon_0\cdotp S} $$

where *ε*_r_, *C*, *d*, *ε*_0_, and *S* are relative dielectric constant, capacitance of the gate dielectric, thickness of the gate dielectric, vacuum dielectric constant, and the area of electrode, respectively.

## Result and Discussion

**Fig. 1:**
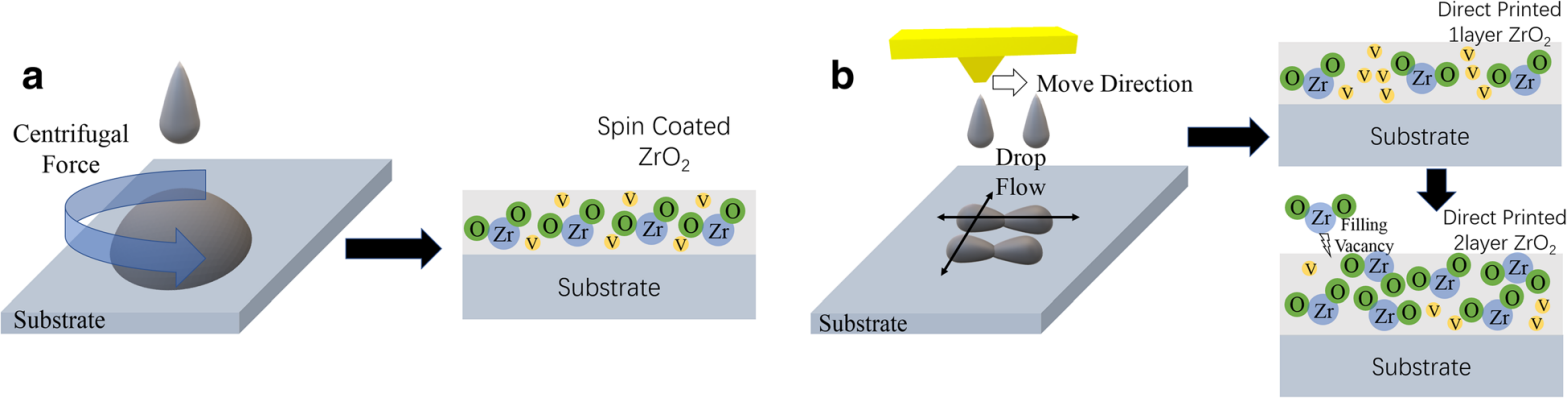
Film formation process of **a** spin coating and **b** direct inkjet printing method

The film formation process of the direct inkjet-printing method compared with the spin-coating method is proposed in Fig. 1. During the spin coating process, droplets are forced to spread uniformly on the whole substrate by centrifugal force [12]. As a consequence, after the annealing process ZrO_2_ molecules are well distributed on the substrate. Meanwhile, the majority of ZrO_2_ molecules are tossed out during the coating process, vacancies occur inside the film. The density of films fabricated by spin coating process are irrelevant to coating parameters for certain precursor [13]. For the inkjet-printing process, the printer moves in a particular direction to leave droplets on the substrate. Droplets merge together at the balance of spreading and shrinking process which is influenced by gravity, surface tension and viscoelasticity of precursor. The film formation process of inkjet printing can be well controlled by optimizing processing parameters of droplet space, jet velocity, ink composition, and substrate temperature [14]. The most important factor is drop space set by the printer and post-treatment process for the substrate. Additional file 1: Figure S1 shows images of the contact angle of printing precursor on ITO substrate with different UV treatment periods and the polarizing microscope of annealed ZrO_2_ films. ZrO_2_ film printed on ITO substrate with 40-s ozone irradiation possesses best morphology. In addition, multiple-layer printing method is efficient in reducing holes in the film by filling vacancies with additional droplets directly printed on the top of the former layer, leading to a more homogeneous film with higher density and less defect [15]. The thickness of films printed 1-layer and 2-layer film is 45 nm and 60 nm, respectively (Additional file 1: Figure S2). Film thickness is not in proportion to printed layers, which explains that the multiple-printing method is not just a thickness accumulation process [16]. In general, the quality of direct-printed ZrO_2_ films can be well controlled by processing parameters. In our experiment, we prepare spin-coated (SC), direct inkjet-printed 1-layer (DP1) and 2-layer (DP2) ZrO_2_ films and IGZO-TFT devices based on these films to investigate the difference in film morphology and electrical property from different film formation processes.

**Fig. 2:**
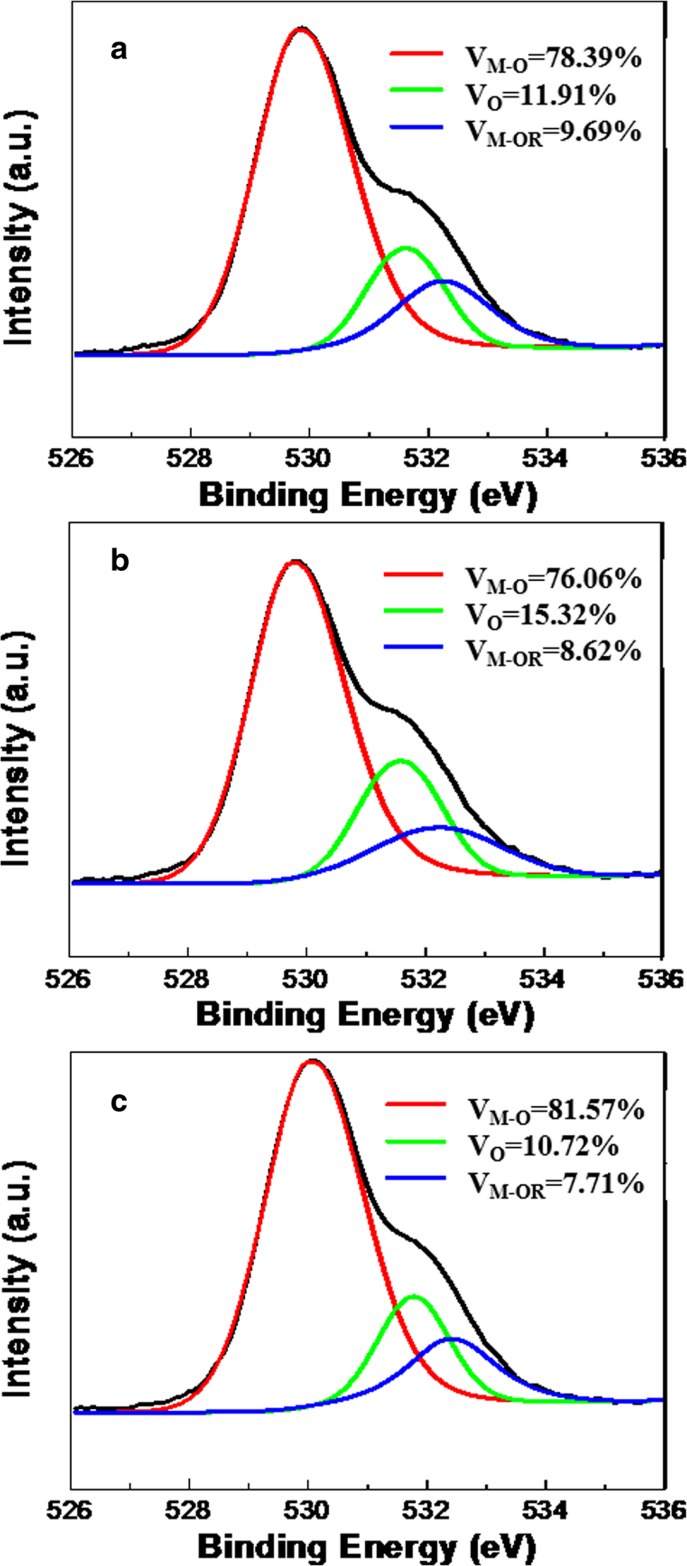
O1s spectrum of **a** SC, **b** DP1-layer, and **c** DP2-layer ZrO_2_ film

Figure 2a–c shows the O1s spectrum of ZrO_2_ film prepared by different methods. We fitted the oxygen 1s peak to a superposition of three peak components. The peaks centered at 529.8 ± 0.2 eV, 531.7 ± 0.2 eV, and 532.1 ± 0.1 eV can be assigned to metal-oxygen bond species (*V*_M-O_), oxygen vacancies (*V*_O_), and weakly bound species (*V*_M-OR_), respectively [17, 18]. The *V*_M-O_ species of the DP2-ZrO_2_ film is 81.57%, which is much higher than the SC-ZrO_2_ and DP1-ZrO_2_. The *V*_O_ species is also the lowest for DP2-ZrO_2_ film. This is consistent with ideas mentioned above: (1) direct inkjet-printing process can obtain ZrO_2_ film with higher density and less oxygen vacancies, and (2) repeated printing process can fill in the holes and traps and reduce vacancies inside the film. AFM measurement was performed to investigate the surface morphology of printed ZrO_2_ film compared with that of spin-coated ZrO_2_ shown in Additional file 1: Figure S3. Spin-coated ZrO_2_ exhibits the smoothest surface with a surface roughness of 0.29 nm, and direct-printed 1-layer and 2-layer ZrO_2_ films are 1.05 nm and 0.67 nm, respectively. Direct-printed ZrO_2_ film possesses a rougher surface owing to the uncontrollable flow of fluid during the film formation process [19]. The remarkable decrease in surface roughness from printing one more layer for direct-printed ZrO_2_ film can be ascribed to fluid printed on the substrate latter fill up the holes of the initial layer to develop a more homogeneous film. The XPS and AFM results show that the inkjet-printing method has a potential in producing higher quality, lower defect dielectric films compared with spin coating method, along with approximate surface roughness which is suitable for TFT fabrication.

**Fig. 3:**
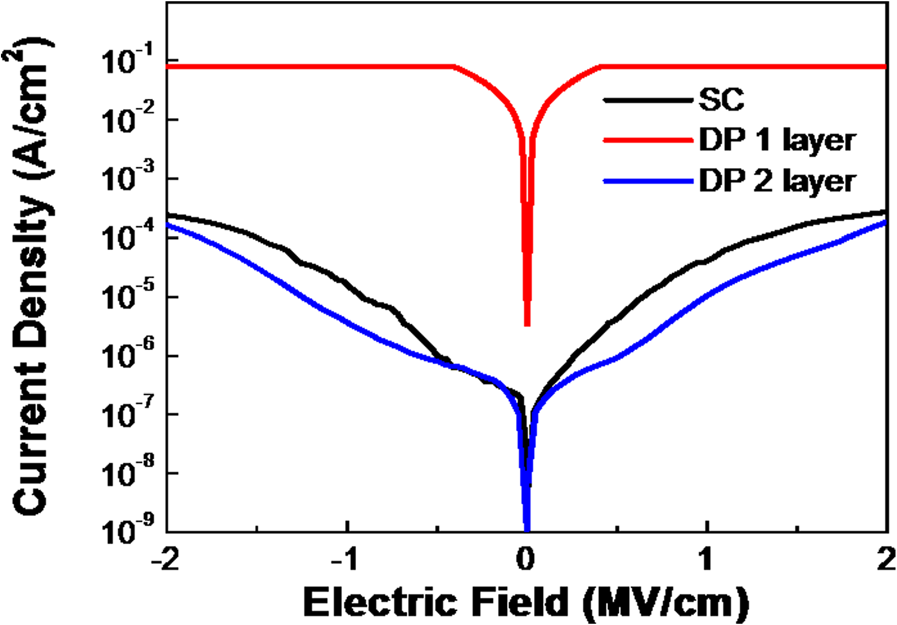
Leakage current density of SC, DP1-layer, and DP2-layer ZrO_2_ film

**Fig. 4:**
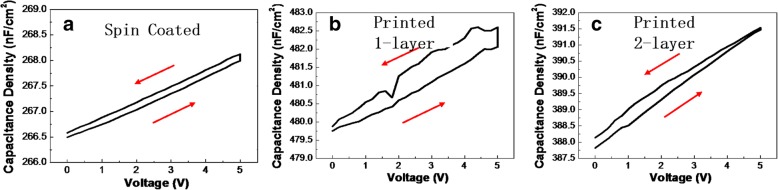
Capacitance density of **a** SC, **b** DP1-layer, and (**c**) DP2-layer ZrO_2_ film

Capacitance-voltage and current-voltage measurements were performed to investigate the electrical properties of SC-ZrO_2_ and DP-ZrO_2_ film using an Al/ZrO_2_/ITO capacitor (metal-insulator-metal) fabricated on glass substrate. We eliminate influence brought by film thickness since they have approximate thickness (60 nm, 45 nm, and 60 nm, respectively). As shown in Fig. 3, DP1-ZrO_2_ film exhibits hardly any insulating property, caused by a large number of vacancies in the film which serve as passage for leakage current. DP2-ZrO_2_ film exhibits the best insulating property, consistent with the result of O 1s spectrum mentioned above. As a result, the leakage current density of DP2-ZrO_2_ film is 2.4 × 10^−5^ A/cm^2^ at 1 MV/cm and the breakdown voltage is over 2.5 MV/cm. In our experiment, printed more layers are similar on the surface roughness and show little improvement in leakage current density compared with printed 2-layer ZrO_2_ film. On the contrary, printing too many layers can easily push the triple line (line of different phase: gas, liquid, solid) moving outward, inducing the nonuniform distribution of precursor ink. Figure 4 shows capacitance-voltage curve of spin-coated and direct-printed ZrO_2_ films. The relative dielectric constant for these three samples is calculated to be 19.2, 20.1, and 18.8 which is close to the reference value (18). For both spin-coated and inkjet-printed ZrO_2_ films, capacitance density increases with voltage hysteresis is observed in both three samples, and it is smallest in SC-ZrO_2_ sample and largest in DP1-ZrO_2_ film. The hysteresis is related to the uniformity and defect state of dielectric film. It confirms that the homogeneity of coating ZrO_2_ film is the best and multiple layer can improve the uniformity of direct inkjet-printing films [20, 21].

**Fig. 5:**
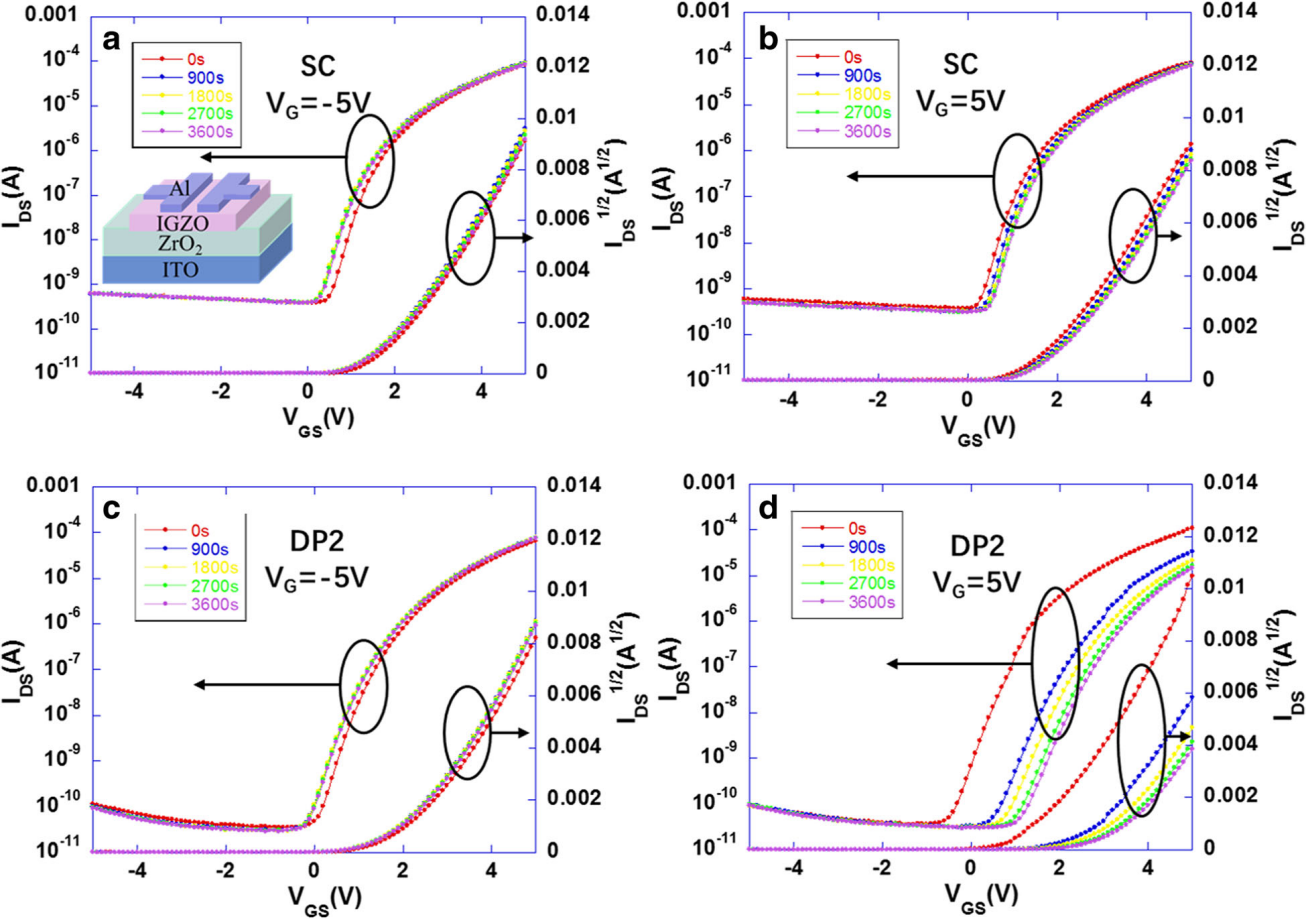
**a** NBS and **b** PBS results of SC-ZrO_2_ IGZO TFT. **c** NBS and **d** PBS results of DP2-ZrO_2_ IGZO TFT

To further study the effect of ZrO_2_ layer fabricated by different ways on TFT performance and gate-bias stability, negative gate-bias stress (NBS) and positive gate-bias stress (PBS) results of IGZO-TFT with both SC-ZrO_2_ and DP2-ZrO_2_ are presented in Fig. 5. Transfer characteristic curves under NBS and PBS were measured by applying a positive (+ 5 V) or negative (− 5 V) bias for 1 h. The DP2-ZrO_2_ IGZO TFT shows better performance at static state with a saturation mobility (*μ*_sat_) of 12.5 cm^2^/V·s, *I*_on_/*I*_off_ radio of 10^6^, and *V*_th_ of 0 V. The SC-ZrO_2_ IGZO TFT exhibits an approximate but lower mobility of 10.2 cm^2^/V·s, worse *I*_on_/*I*_off_ radio of 2 × 10^5^, and higher off-state current (*I*_off_), mainly due to an increase of channel leakage by larger amount of oxygen vacancies (*V*_O_) in the dielectric film. The *V*_th_ shift of IGZO TFT with both SC-ZrO_2_ and DP2-ZrO_2_ under NBS measurements is negligible. The negative *V*_th_ shift of oxide TFTs under NBS is generally caused by the hole trapping or charge injection since the ionized oxygen vacancies can migrate to the semiconductor/insulator interface under the negative gate bias field. The NBS results indicate that either SC-ZrO_2_ or DP2-ZrO_2_ film has a favorable contact with IGZO [22, 23]. However, unlike SC-ZrO_2_ IGZO TFT which exhibits a *V*_th_ shift of 0.4 V after applying PBS for 1 h, the DP2-ZrO_2_ IGZO TFT shows a severe degeneration of performance and large *V*_th_ shift of 1.2 V under PBS test. The results of ZrO_2_-IGZO TFTs under PBS test are summarized in Table 1. Since the *V*_th_ shift of oxide TFTs under PBS test is generally caused by the diffusion of absorbed water or oxygen molecules, we can assume that the backchannel of DP2-ZrO_2_ IGZO TFT is more sensitive to atmospheric environment under PBS test [24, 25].

**Table 1:** The summary of mobility, *I*_on_/*I*_off_ ratio, and *V*_th_ during PBS test of spin-coated and direct-printed ZrO_2_ TFT

SC-TFT	Mobility (cm^2^/V·s)	*I*_on_/*I*_off_ ratio	*V*_th_ (V)
Time (s)			
0	10.2	2.0 × 10^5^	−0.2
900	9.9	1.8 × 10^5^	0.0
1800	9.8	1.7 × 10^5^	0.1
2700	9.6	1.6 × 10^5^	0.1
3600	9.6	1.6 × 10^5^	0.2
DP2-TFT	Mobility (cm^2^/V·s)	*I*_on_/*I*_off_ ratio	*V*_th_ (V)
Time (s)			
0	12.5	1.0 × 10^6^	−0.6
900	8.8	3.0 × 10^5^	0.3
1800	7.8	1.4 × 10^5^	0.5
2700	7.2	1.0 × 10^5^	0.6
3600	6.8	9.5 × 10^4^	0.7

**Fig. 6:**
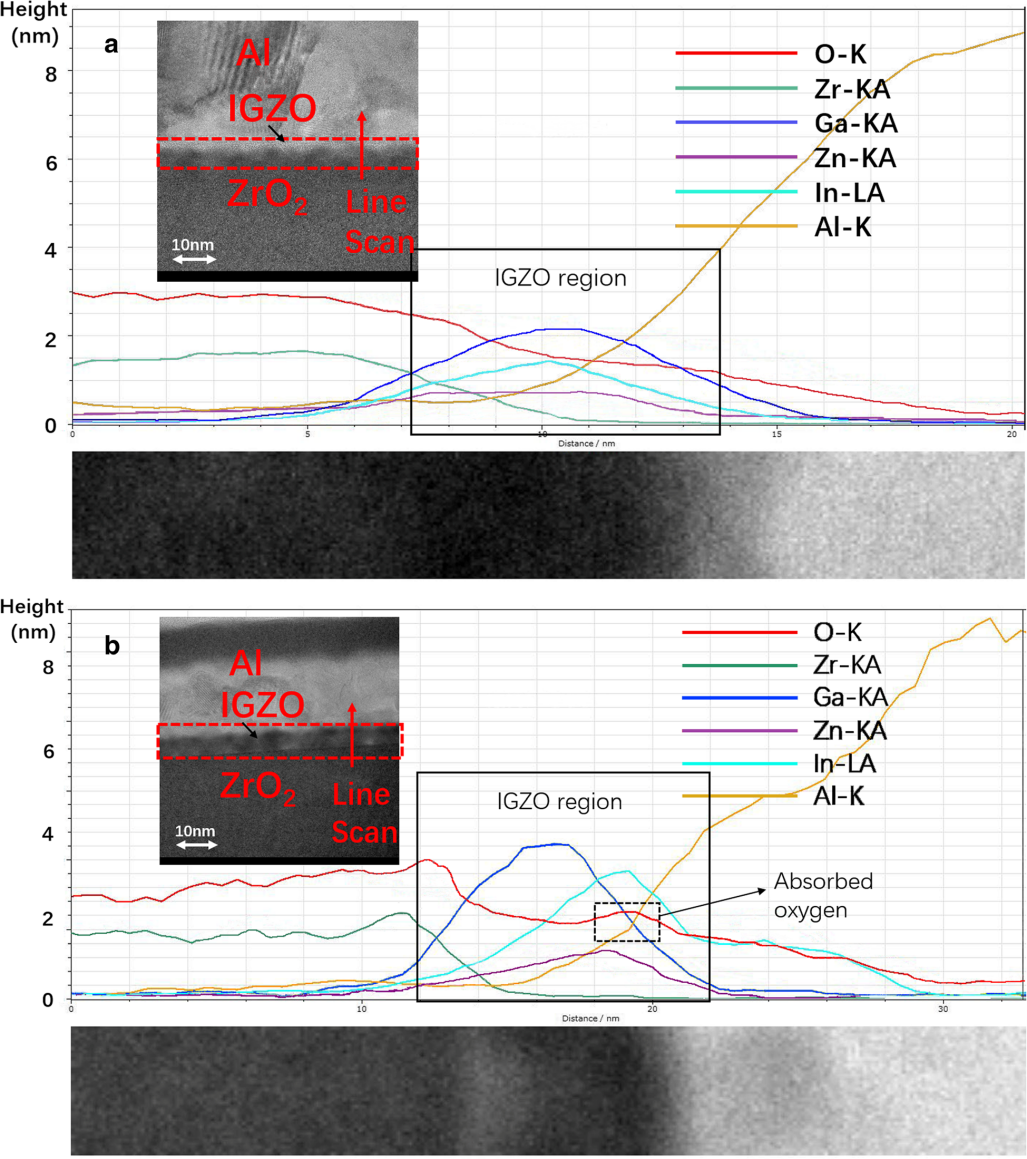
**a** TEM image and EDS line scanning of SC-ZrO_2_ IGZO TFT. **b** TEM image and EDS line scanning of DP2-ZrO_2_ IGZO TFT

To investigate the degeneration and V_th_ shift under PBS test for ZrO_2_-IGZO TFT, the cross-sectional transmission electron microscopy (TEM) images and EDS line scan were measured to analyze the element distribution. From the cross-sectional TEM images shown in Fig. 6**a** and **b**, a structure of the Al/IGZO/ZrO_2_ investigated in this paper was presented. From the high-resolution TEM images of the channel region for both SC-ZrO_2_ IGZO TFT and DP2-ZrO_2_ IGZO TFT, a nearly 8-nm-thick IGZO layer can be obviously observed, which can be proved by the distribution of the In (Ga, Zn) element in EDS line scanning results. Meanwhile, for both SC-ZrO_2_ IGZO TFT and DP2-ZrO_2_ IGZO TFT, the ZrO_2_ layer exhibits an amorphous structure which is beneficial to low-leakage current density. It is obvious that from the line scanning result, Al element diffuse into the IGZO layer, which may be caused by impact during the Al sputtering process. Furthermore, the ratio of Zr and O element is approximately 1:2, which demonstrates that pure ZrO_2_ was formed after the annealing process. Uniform distribution of In, Ga, Zn, and Zr elements are also obtained in the IGZO layer for SC-ZrO_2_ IGZO TFT, indicating a homogeneous structure of ZrO_2_ and IGZO film was established during sputtering and the post-annealing process [19]. But for DP2-ZrO2 IGZO TFT, In, Ga, Zn, O and Zr are in irregular distribution. From Fig. 6(b), we can see the Zr element along with the O element is concentrated at the interface of the dielectric and active layer. And it totally coincided with the analysis of the film formation process of multiple-layer printing method. During the multiple-printing process, the precursor printed latter on the substrate partly fills the vacancies, and the majority of droplets are accumulating at the top [26]. Moreover, the segregation of In and Zn element at the backchannel of the IGZO layer is observed in the IGZO layer of printed ZrO_2_-TFT. Since the proportion of the Zn element is minimum in our experiment, the electrical performance of IGZO TFT is determined by the In and Ga element. The formation of an In-rich region at the Al/IGZO interface can be concluded as follows: during the annealing process of the IGZO layer which aims to eliminate the defect state of IGZO, there was a redistribution of each element. O atoms were “taken away” from In and Zn elements since they have lower oxygen bond dissociation energy than the Zr element, pushing them away from the dielectric/semiconductor interface. The elementary substance of In and Zn elements are unstable so they recombined with oxygen absorbed at the back channel, which can be proved by the EDS scanning [27–29]. The In-rich region with absorbed water molecules and oxygen is the reason for a large *V*_th_ shift under PBS test.

**Fig. 7:**
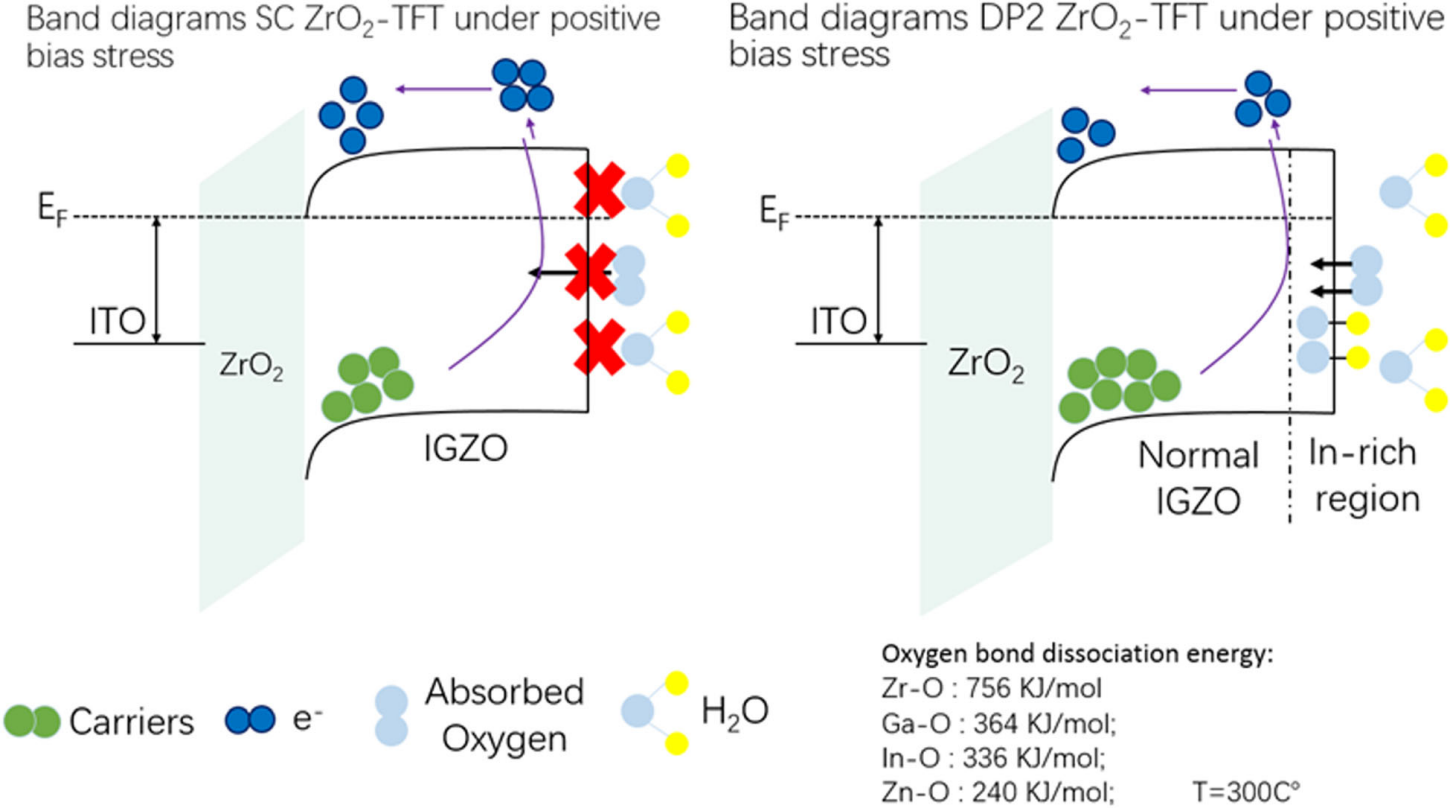
Band diagrams SC-ZrO_2_ TFT and DP2-ZrO_2_ TFT under positive bias stress

In order to conceptually depict the mechanism of the degenerated performance and *V*_th_ shift under positive bias stress for IGZO TFT, schematic band diagrams of TFT for spin-coated ZrO_2_ and inkjet-printed ZrO_2_ are shown in Fig. 7. DP2-ZrO_2_ TFT can accumulate more carriers than SC-ZrO_2_ TFT at static state due to a better insulating property, but under positive bias stress, most carriers are exhausted by acceptor-like molecules like water and oxygen in the atmosphere. In general, hydrogen, oxygen, and H_2_O molecules will incorporate into the IGZO thin film due to diffusion in the backchannel. Afterwards, the hydrogen will react with oxygen and generate oxygen-hydroxide bonds and consume electrons which results in degenerated performance under positive bias stress. Meanwhile, the adsorbed O_2_ and H_2_O molecules act as an acceptor-like trap that can capture electrons from conduction band, leading to the positive *V*_th_ shift after PBS [30]. The degenerated performance and *V*_th_ shift are unstable and it can recover after hours under an ambient atmosphere. Owing to different oxygen bond dissociation energies of Zr-Oxide (756 kJ/mol), Ga-Oxide (364 kJ/mol), In-Oxide (336 kJ/mol), and Zn-Oxide (240 kJ/mol) [31], O atoms are more likely to combine with the Zr element due to large oxygen bond dissociation energies. The In and Zn element pushed away from ZrO_2_/IGZO interface to the backchannel absorb oxygen in the environment. For IGZO TFT using direct inkjet-printed ZrO_2_ as gate insulator, large amounts of hydrogen, oxygen, and H_2_O molecules “consume” the electrons when applying positive bias stress, leading to degeneration of device performance. Methods including introducing a passivation layer in the top of source/drain electrode for bottom gate structure, using top gate structure, and introducing an interface modification layer between the dielectric and semiconductor layer are effective ways to improve PBS for a solution-processed TFT device, which is interesting and will be carried out in our further research.

## Conclusion

In conclusion, we fabricated a high-quality direct inkjet-printed ZrO_2_ gate insulator using multiple-layer printing method without extra patterning technology, which is suitable for large-size printing fabrication process. The film formation process demonstrates that ZrO_2_ film fabricated by direct inkjet-printing process obtains a denser structure compared with the spin coating process, but the homogeneity is worse because of the uncontrollable fluid flow of precursor ink. XPS results indicate printed 2-layer ZrO_2_ film possesses the highest percentage of M-O-M species (*V*_M-O_) and lowest oxygen vacancies (*V*_O_), reflecting in a low leakage current density. Capacitance-voltage curve of DP2-ZrO_2_ film shows a slight hysteresis, which is similar with SC-ZrO_2_. As a result, DP2-ZrO_2_ film exhibits a relatively low leakage current density of 2.4 × 10^−5^A/cm^2^ at 1 MV/cm and a breakdown voltage over 2 MV/cm; TFT device based on DP2-ZrO_2_ exhibited a saturation mobility of 12.4 cm^2^/Vs, an *I*_on_/*I*_off_ ratio of 10^6^, a turn on voltage of 0 V, and a 1.2-V *V*_th_ shift after 1 h PBS test. The segregation of the In element at the backchannel of the IGZO layer observed in TEM image and EDS scan can be responsible for larger V_th_ shift during PBS test due to the adsorbed O_2_ and H_2_O molecules which act as acceptor-like trap that can capture electrons from conduction band. This article presents the advantages of direct inkjet-printing technology and investigates the dielectric property for solution-processed oxide insulator used in oxide TFT device. It demonstrates that DP2-ZrO_2_ has a denser structure with less oxygen vacancies, but poor stability under PBS caused by element diffusion. It is promising for direct inkjet-printing technology to be applied in mass production since its low cost and high performance after improving its stability.

## Additional file

Additional file 1: **Figure S1.** Images of contact angle of oxide precursor on ITO substrate for different ozone UV treatment period: (a) 20s, (b) 40s, and (c) 60s and polarizing microscope of annealed ZrO_2_ film on ITO substrate for different UV treatment period (d) 20s, (e) 40s, and (f) 60s, respetively. Figure S2. Step profiler images of direct-printed (a) 1-layer and (b) 2-layer ZrO_2_ films. Figure S3. AFM images of ZrO_2_ film prepared by (a) spin coating, (b)direct-printed 1 layer, and (c) direct-printed 2 layers. (DOCX 2208 kb)

## Abbreviations

2MOE: Methoxyethanol (solvent)
AFM: Atomic force microscope
Al: Aluminum
DP1/2: Direct-printed 1/2 layer
EDS: Electronic differential system
H_2_O: Water molecule
IGZO: Indium gallium zinc oxide (oxide semiconductor)
ITO: Indium tin oxide (electrode)
O 1s: Oxide 1s atomic orbital
O_2_: Oxygen molecule
PBS/NBS: Positive/negative bias stress (test mode)
SC: Spin coated
SiN_*x*_: Silicon nitride (dielectric)
SiO_2_: Silicon Dioxide (dielectric)
TEM: Transmission electron microscope
TFT: Thin-film transistor
UV: Ultraviolet
*V*_M-O_: Percentage of metal-oxide bond
*V*_M-OR_: Percentage of metal-organics bond
*V*_O_: Percentage of oxide vacancy bond
*V*_th_: Threshold voltage
XPS: X-ray photoelectron spectroscopy
ZrO_2_: Zirconia (oxide dielectric)
ZrOCl_2_·8H_2_O: Zirconium oxychloride octahydrate (material)
