# Supplementary material for: Investigation of direct inkjet-printed versus spin-coated ZrO2 for sputter IGZO thin film transistor
Source: Nanoscale Res Lett. 2019 Mar 5;14:80. doi: 10.1186/s11671-019-2905-2 (PMC6401082; doi:10.1186/s11671-019-2905-2)
Supplement: Supplementary file 1 — Figure S1. Images of contact angle of oxide precursor on ITO substrate for different ozone UV treatment period: (a) 20s, (b) 40s, and (c) 60s and polarizing microscope of annealed ZrO2 film on ITO substrate for different UV treatment period (d) 20s, (e) 40s, and (f) 60s, respetively. Figure S2. Step profiler images of direct-printed (a) 1-layer and (b) 2-layer ZrO2 films. Figure S3. AFM images of ZrO2 film prepared by (a) spin coating, (b)direct-printed 1 layer, and (c) direct-printed 2 layers. (DOCX 2208 kb) [file 11671_2019_2905_MOESM1_ESM.docx]

**Supplementary material**

**Investigation of direct inkjet-printed versus spin-coated ZrO_2_ for sputter IGZO thin film transistor**

*Wei Cai^1^, Honglong Ning^1a^, Zhennan Zhu^1^, Jinglin Wei^1^, Shangxiong Zhou^1^, Rihui Yao^1b^, Zhiqiang Fang^2^, Xiuqi Huang^3^**, Xubing Lu^4^, Junbiao Peng^1^*

*^1^Institute of Polymer Optoelectronic Materials & Devices, State Key Laboratory of Luminescent Materials & Devices, South China University of Technology, P.R. China*

**Corresponding author: ninghl@scut.edu.cn;* [*yaorihui@scut.edu.cn*](mailto:yaorihui@scut.edu.cn)

*^2^State Key Laboratory of Pulp and Paper Engineering, South China University of Technology, Guangzhou 510640, China*

*^3^Gu’an New Industry Demonstration Zone, Langfang, Hebei, Postal code 065500, P.R China*

*^4^Institute for Advanced Materials and Guangdong Provincial Key Laboratory of Quantum Engineering and Quantum Materials, South China Normal University, Guangzhou 510006, China*

For ozone UV treatment process, a 100W UV lamp with 250nm wavelength was used to irradiate the ITO substrate cleaned by isopropyl alcohol and deionized water. Polarizing microscope images were obtained from Nikon Eclipse E600 POL to observe the surface appearance of dielectric films. The surface tension and contact angle of inks were obtained using a contact angle analyzer (Biolin Scientific, Theta Lite 101) and a viscometer (Brookfield, DV-I+), respectively. Surface profiles and morphology images were characterized by Veeco NT 9300. The surface morphology and roughness were observed using atomic force microscopy (AFM, Asylum Research) in tapping mode.


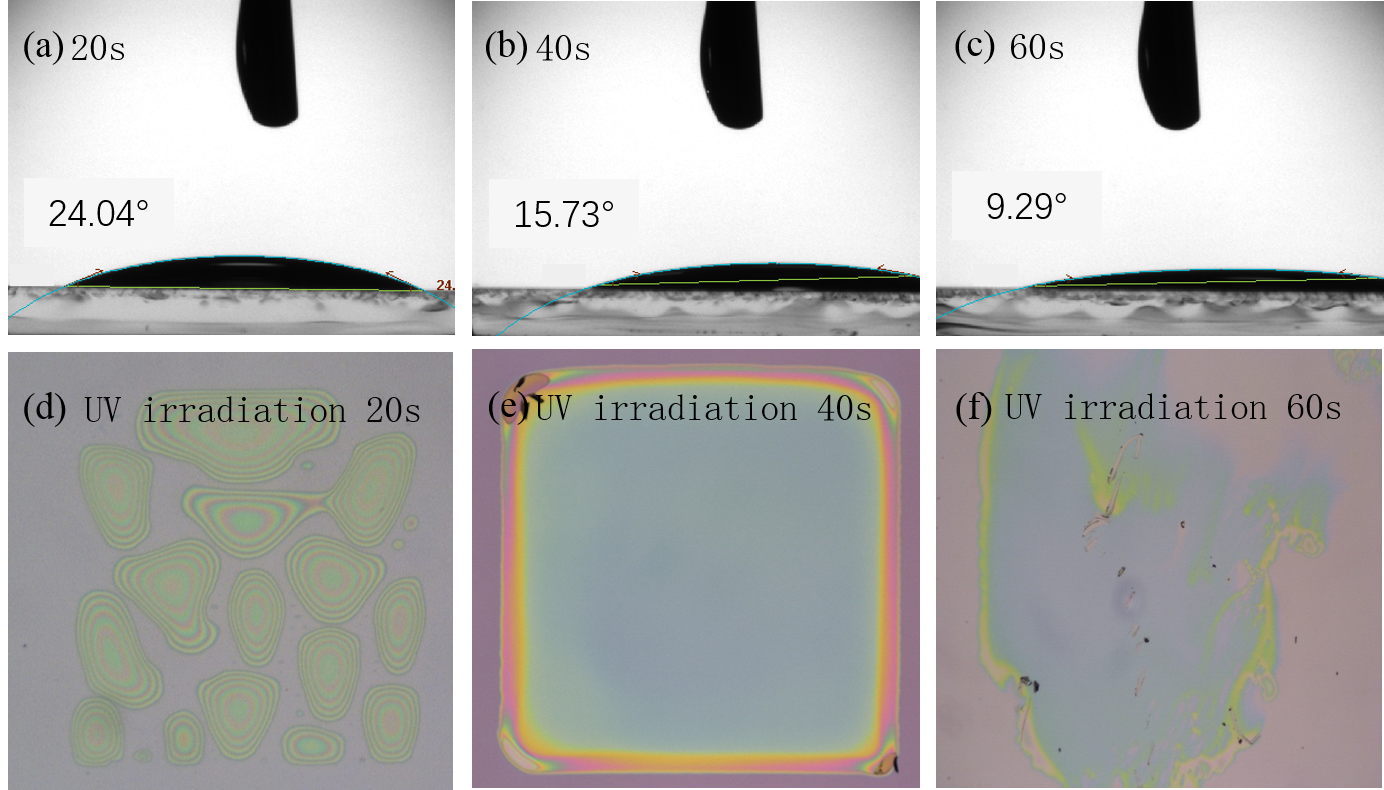


Figure S1. Images of contact angle of oxide precursor on ITO substrate for different ozone UV treatment period: (a)20s; (b)40s and (c)60s and polarizing microscope of annealed ZrO_2_ film on ITO substrate for different UV treatment period (d)20s; (e)40s and (f)60s, respetively

Figure S1 shows Images of contact angle on ITO substrate for different UV treatment period and the polarizing microscope of annealed ZrO_2_ film. Figure S1(a-c) shows contact angle varies from 24.04° to 15.73° and 9.29° as ozone UV irradiation time increasing from 20s to 40s. Figure S1(d) shows that annealed oxide precursor printing on the substrate cannot form continuous film after ozone UV treatment for 20s. And from Figure S1(e) we can see a uniform ZrO_2_ film formed on the substrate except for boundary area caused by coffee ring effect which cannot be entirely eliminated. As the UV treatment time for ITO substrate increases to 60s, the spreading of oxide precursor on the substrate becomes uncontrollable, resulting in a heterogeneous film seen in Figure S1(f). The leakage passage always generates at the weak side of dielectric film so a heterogeneous film will undoubtedly fail at leakage current test. From figure S1 we can make a conclusion the spreading of precursor is very sensitive to the UV irradiation on the ITO substrate. UV irradiation can “clean” the substrate reflecting in the change of contact angle, and excessive irradiation can ruin the spreading process as well.


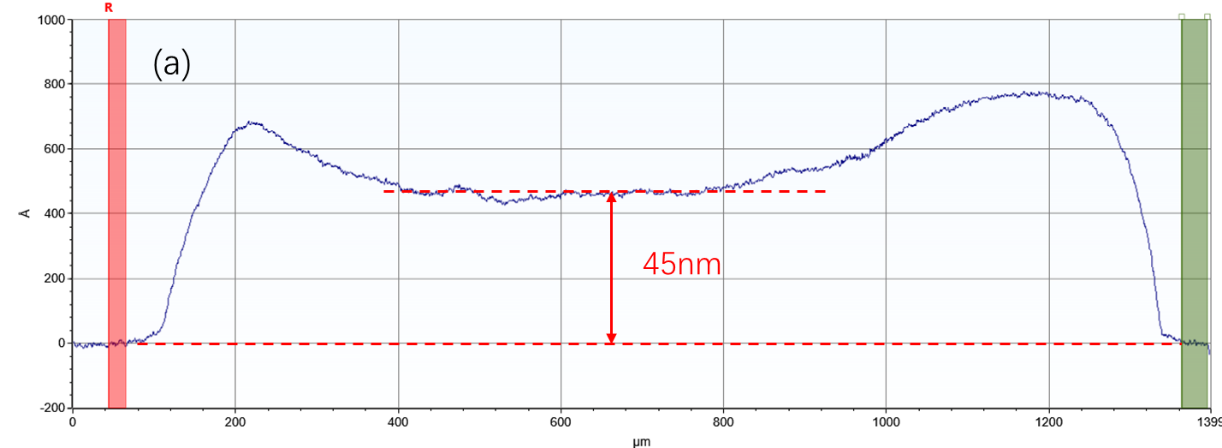


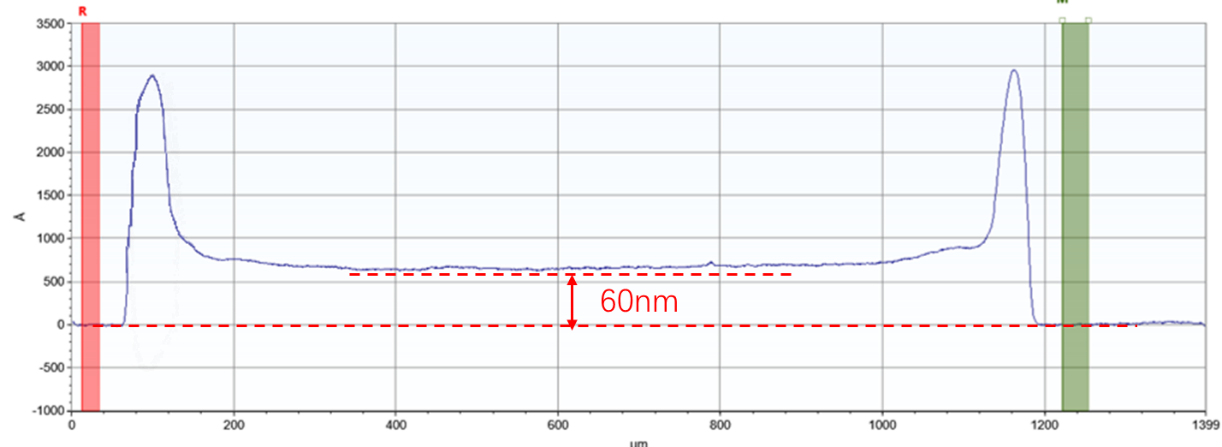


Figure S2. Step profiler Images of direct printed (a) 1 layer and (b)2 layers ZrO_2_ films

The thickness of films printed 1 layer and 2 layers is 45nm and 60nm respectively, detected by step profiler. Film thickness is obviously not in proportion to printed layers. The coffee ring effect exacerbates as increasing printing layers, but there is still a wide region which is flat enough for semiconductor fabrication.


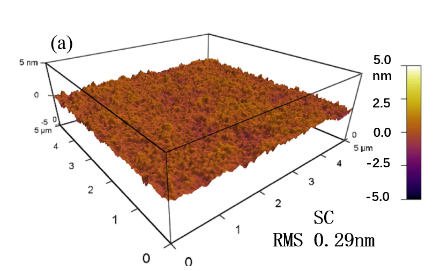


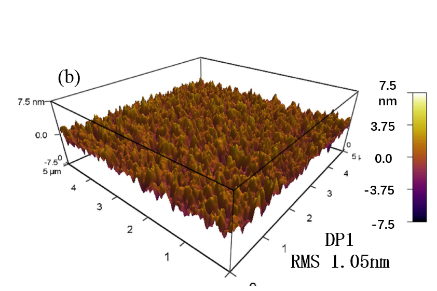


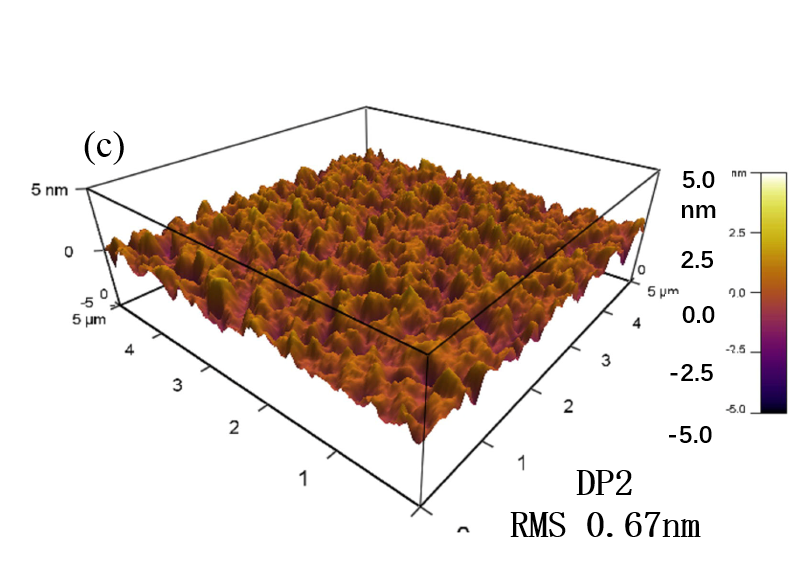


Figure S3. AFM Images of ZrO_2_ film prepared by (a)spin coated; (b)direct printed 1 layer and (c) direct printed 2 layers

Figure S3 shows AFM measurement of ZrO_2_ film prepared by spin coating method compared with direct printing method. Spin coated ZrO_2_ exhibits the smoothest surface with a surface roughness of 0.29nm, that for direct printed 1 layer ZrO_2_ film and 2 layers is 1.05nm and 0.67nm, respectively. Better surface morphology can reduce defects in semiconductor/dielectric interface, results in better device electrical performance including mobility and bias stress stability. Printed ZrO_2_ films obtain worse surface roughness due to the uncontrollable fluid flow of precursor, and it can be improved by multiple printing process.
